# Cyanobacterial KnowledgeBase (CKB), a Compendium of Cyanobacterial Genomes and Proteomes

**DOI:** 10.1371/journal.pone.0136262

**Published:** 2015-08-25

**Authors:** Arul Prakasam Peter, Karthick Lakshmanan, Shylajanaciyar Mohandass, Sangeetha Varadharaj, Sivasudha Thilagar, Kaleel Ahamed Abdul Kareem, Prabaharan Dharmar, Subramanian Gopalakrishnan, Uma Lakshmanan

**Affiliations:** 1 National Facility for Marine Cyanobacteria, Sub-Distributed Bioinformatics Centre (sponsored by Department of Biotechnology, Govt. of India), Department of Marine Biotechnology, School of Marine Sciences, Bharathidasan University, Tiruchirappalli, Tamil Nadu, India; 2 Department of Environmental Biotechnology, Bharathidasan University, Tiruchirappalli, Tamil Nadu, India; 3 Department of Botany, Jamal Mohammed College, Tiruchirappalli, Tamil Nadu, India; Nazarbayev University, KAZAKHSTAN

## Abstract

Cyanobacterial KnowledgeBase (CKB) is a free access database that contains the genomic and proteomic information of 74 fully sequenced cyanobacterial genomes belonging to seven orders. The database also contains tools for sequence analysis. The Species report and the gene report provide details about each species and gene (including sequence features and gene ontology annotations) respectively. The database also includes cyanoBLAST, an advanced tool that facilitates comparative analysis, among cyanobacterial genomes and genomes of *E*. *coli* (prokaryote) and *Arabidopsis* (eukaryote). The database is developed and maintained by the Sub-Distributed Informatics Centre (*sponsored by* the Department of Biotechnology, *Govt*. *of India*) of the National Facility for Marine Cyanobacteria, a facility dedicated to marine cyanobacterial research. CKB is freely available at http://nfmc.res.in/ckb/index.html.

## Introduction

Cyanobacteria comprise over 1,600 species with various morphologies and species-specific characteristics, such as cell movement, cell differentiation, and nitrogen fixation [1]. These are the only known oxygenic photosynthetic prokaryotic organisms that inhabit a wide range of ecological habitats (e.g., extreme cold, extreme hot, marine, fresh water, and terrestrial) and exhibit symbiotic associations with other living organisms. These primitive oxygenic Gram negative bacteria are widely used as a valuable model to study the mechanism of carbon fixation and helpful for evolutionary biologists to understand the endosymbiotic theory, as they are considered as the origin of chloroplast. Since these ancient life forms play a major role in many biogeochemical cycles of the global ecological system, they serve as a study material in diverse fields of life-science research [2].

Cyanobacteria are well-known for the formation of toxic cyanobacterial water blooms in freshwater, brackish and coastal marine ecosystems, which are of vital ecological and human health concerns [3]. However, in recent times, these organisms have captured the attention of the researchers worldwide because of their capability of producing prolific bioactive natural products as secondary metabolites, which are of great economic and medical value [4–6].

The National Facility for Marine Cyanobacteria (*Sponsored by* the Department of Biotechnology, *Govt*. *of India*) is dedicated to cyanobacterial research, especially marine cyanobacteria. One of the principal foci of the facility is to build a dedicated knowledge base for cyanobacteria. The increasing number of completely sequenced cyanobacterial genomes provides wide opportunities for understanding the metabolic organization of the cyanobacterial species in diverse environments. Here we introduce the Cyanobacterial KnowldegeBase (CKB), a freely accessible, comprehensive database resource covering information pertaining to 74 completely sequenced cyanobacterial species. The database also includes an informative tool called cyanoBLAST, which helps in comparative analysis between cyanobacterial genomes and the genomes of pro- and eu-karyote, such as *E*. *coli* and *Arabidopsis*.

## Results and Discussion

### Organisms

**Fig 1:**
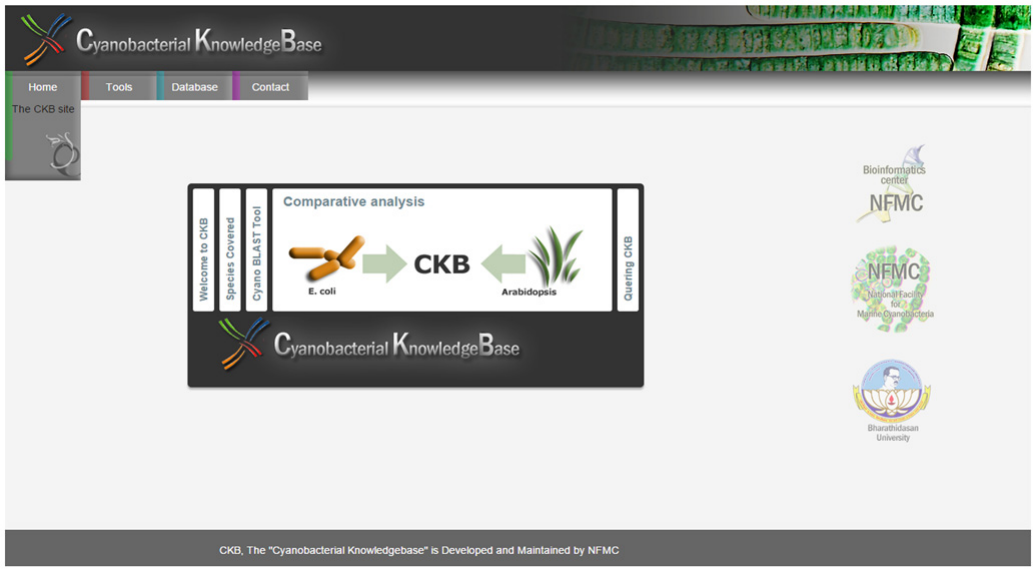
CKB web interface.

Seventy-four fully sequenced genomes of seven orders are currently included in the CKB database. This comprises 12 species of Chroococcales, 1 of Chroococcidiopsidales, 2 of Gloeobacteriales, 12 of Nostocales, 7 of Oscillatoriales, 2 of Pleurocapsales and 38 of Synechococcales. The web user interface of CKB is shown in (Fig 1) and the complete list of the species exists in the CKB is given in Table 1.

**Table 1:** Order wise complete list of species mentioned in CKB. The table provides information related to order, Morphology (Morph.-U: Unicellular, F: Filamentous and F,H: Filamentous Heterocystous), number of chromosomes (Chr.), number of plasmids (Pla.), genome size (Size, MB), GC %, the number of genes (Genes), number of proteins (Proteins), and Biological Resource Centers (BRCs) from which live specimens can be available for each species.

Order	Organism	Morph.	Chr.	Pla.	Size, MB	GC %	Genes	Proteins	BRCs
**Chroococcales**	*Cyanobacterium aponinum* PCC 10605	U	1	0	4.72	60.5	4562	4507	PCC
	*Cyanobacterium stanieri* PCC 7202	U	1	0	4.66	62	4482	4430	PCC
	*Cyanothece* sp. ATCC 51142	U	1	6	7.06	38.8	6258	5838	ATCC
	*Cyanothece* sp. PCC 7424	U	2	3	5.31	38.1	4797	4511	PCC
	*Cyanothece* sp. PCC 7425	U	2	3	7.11	41.4	5813	5710	ATCC; PCC
	*Cyanothece* sp. PCC 7822	U	1	3	6.96	39.8	5841	5535	PCC
	*Cyanothece* sp. PCC 8801	U	1	0	7.02	42.2	6250	5950	PCC
	*Cyanothece* sp. PCC 8802	U	1	3	7.61	42.2	6738	6229	PCC
	*Dactylococcopsis salina* PCC 8305	U	1	2	5.49	38.3	5380	3651	PCC
	*Gloeocapsa* sp. PCC 7428	U	1	5	9.06	41.3	7164	6689	ATCC; PCC
	*Halothece* sp. PCC 7418	U	1	0	6.33	40.4	5538	5237	PCC
	*Microcystis aeruginosa* NIES-843	U	1	6	7.21	41.2	6213	6129	NIES
**Chroococcidiopsidales**	*Chroococcidiopsis thermalis* PCC 7203	U	1	2	6.72	41.5	5687	5449	ATCC; PCC
**Gloeobacteriales**	*Gloeobacter kilaueensis* JS1	U	1	2	8.73	37.5	6946	6644	NA
	*Gloeobacter violaceus* PCC 7421	U	1	9	8.36	47	8571	8383	ATCC; PCC
**Nostocales**	*Anabaena cylindrica* PCC 7122	F, H	1	0	6.79	44.3	6676	6630	ATCC; PCC
	*Anabaena* sp. 90	F, H	1	2	6.76	45.6	6426	5945	NA
	*Anabaena variabilis* ATCC 29413	F, H	1	8	5.62	40.2	5059	4752	ATCC
	*Calothrix* sp. PCC 630	F, H	1	1	4.18	35	3614	3431	ATCC; PCC
	*Calothrix* sp. PCC 7507	F, H	1	0	3.16	38.7	2941	2837	ATCC; PCC
	*Cylindrospermum stagnale* PCC 7417	F, H	1	0	3.34	68.7	3437	3280	ATCC; PCC
	*Nostoc azollae* 0708	F, H	2	4	5.46	38	5364	5303	NA
	*Nostoc punctiforme* PCC 73102	F, H	1	6	6.55	38.5	5942	5710	ATCC; PCC
	*Nostoc* sp. PCC 7107	F, H	1	3	5.79	50.7	5507	5327	ATCC; PCC
	*Nostoc* sp. PCC 7120	F, H	1	6	7.84	39.9	7042	6642	ATCC; PCC
	*Nostoc* sp. PCC 7524	F, H	1	3	4.79	39.8	4619	4367	ATCC; PCC
	*Rivularia* sp. PCC 7116	F, H	1	4	4.8	39.8	4700	4444	PCC
**Oscillatoriales**	*Arthrospira platensis* NIES-39	F	1	0	3.78	42.4	3684	3337	NIES
	*Crinalium epipsammum* PCC 9333	F	1	0	4.68	58.5	3912	3815	PCC
	*Geitlerinema* sp. PCC 7407	F	1	4	5.88	43.4	5304	5011	ATCC; PCC
	*Microcoleus* sp. PCC 7113	F	1	0	4.18	42.9	3920	3708	PCC
	*Oscillatoria acuminata* PCC 6304	F	1	0	5.13	43.9	4654	4228	ATCC; PCC
	*Oscillatoria nigro-viridis* PCC 7112	F	1	8	7.97	46.2	6821	6441	PCC
	*Trichodesmium erythraeum* IMS101	F	1	0	5.84	42.3	6364	6312	NCMA
**Pleurocapsales**	*Pleurocapsa* sp. PCC 7327	U	1	2	7.8	47.6	6100	5796	ATCC; PCC
	*Stanieria cyanosphaera* PCC 7437	U	1	5	8.27	45.8	7006	6360	ATCC; PCC
**Synechococcales**	*Acaryochloris marina* MBIC11017	U	1	1	4.89	46.2	4014	3854	NA
	*Chamaesiphon minutus* PCC 6605	U	1	0	2.7	55.5	2581	2522	PCC
	*Cyanobium gracile* PCC 6307	U	1	1	2.74	55.5	2715	2662	PCC
	*Synechococcus elongatus* PCC 6301	U	1	0	2.61	52.4	2944	2892	PCC
	*Synechococcus elongatus* PCC 7942	U	1	0	2.51	59.2	2756	2645	ATCC; PCC
	*Synechococcus* sp. CC9311	U	1	0	2.23	54.2	2357	2306	NCMA
	*Synechococcus* sp. CC9605	U	1	0	3.05	58.5	2942	2862	NCMA
	*Synechococcus* sp. CC9902	U	1	0	2.93	60.2	2897	2760	NCMA
	*Synechococcus* sp. JA-2-3B'a(2–13)	U	1	1	3.72	48.5	3794	3545	NA
	*Synechococcus* sp. JA-3-3Ab	U	1	6	3.41	49.2	3238	3187	NA
	*Synechococcus* sp. PCC 6312	U	1	2	3.58	40.6	3666	3318	ATCC; PCC
	*Synechococcus* sp. PCC 7002	U	1	0	2.22	60.8	2581	2533	ATCC; PCC
	*Synechococcus* sp. PCC 7502	U	1	0	2.37	60.2	2586	2533	PCC
	*Synechococcus* sp. RCC307	U	1	0	2.43	59.4	2581	2519	RCC
	*Synechococcus* sp. WH 7803	U	1	0	3.57	47.7	3219	3170	NCMA
	*Synechococcus* sp. WH 8102	U	1	4	3.95	47.3	3625	3575	NCMA
	*Synechocystis* sp. PCC 6803	U	1	7	3.95	47.3	3610	3561	PCC
	*Synechocystis* sp. PCC 6803	U	1	0	3.57	47.7	3218	3169	PCC
	*Synechocystis* sp. PCC 6803	U	1	0	3.57	47.7	3217	3168	PCC
	*Synechocystis* sp. PCC 6803 substr. GT-I	U	1	0	3.57	47.7	3217	3168	PCC
	*Synechocystis* sp. PCC 6803 substr. PCC-N	U	1	0	2.59	53.9	2525	2476	PCC
	*Synechocystis* sp. PCC 6803 substr. PCC-P	U	1	0	2.52	53.8	2400	2231	PCC
	*Thermosynechococcus elongatus* BP-1	U	1	0	7.75	34.1	5126	4451	NA
	*Thermosynechococcus* sp. NK55	U	1	2	6.69	44.4	6033	5752	NA
	*Leptolyngbya* sp. PCC 7376	U	1	0	4.99	45.2	4665	4268	ATCC; PCC
	*Pseudanabaena* sp. PCC 7367	U	1	5	5.54	36.3	5041	4781	PCC
	*Prochlorococcus marinus* str. AS9601	U	1	0	1.67	31.3	1965	1920	NCMA
	*Prochlorococcus marinus* str. MIT 9211	U	1	0	1.69	38	1900	1854	NA
	*Prochlorococcus marinus* str. MIT 9215	U	1	0	1.74	31.1	2054	1982	NCMA
	*Prochlorococcus marinus* str. MIT 9301	U	1	0	1.64	31.3	1962	1906	NCMA
	*Prochlorococcus marinus* str. MIT 9303	U	1	0	2.68	50	3136	2997	NCMA
	*Prochlorococcus marinus* str. MIT 9312	U	1	0	1.71	31.2	1856	1810	NCMA
	*Prochlorococcus marinus* str. MIT 9313	U	1	0	2.41	50.7	2330	2269	NCMA
	*Prochlorococcus marinus* str. MIT 9515	U	1	0	1.7	30.8	1964	1905	NCMA
	*Prochlorococcus marinus* str. NATL1A	U	1	0	1.86	35	2250	2193	NCMA
	*Prochlorococcus marinus* str. NATL2A	U	1	0	1.84	35.1	2228	2162	NCMA
	*Prochlorococcus marinus* subsp. *marinus* str. CCMP1375	U	1	0	1.75	36.4	1930	1882	NCMA
	*Prochlorococcus marinus* subsp. *pastoris* str. CCMP1986	U	1	0	1.66	30.8	1762	1717	NCMA

The Biological Resource Centers (BRCs) listed (with hyperlinks) includes 1. ATCC (American Type Culture Collection), 2. PCC (Pasteur Culture Collection of Cyanobacteria), 3. NIES (National Institute for Environmental Studies), 4. NCMA (National Center for Marine Algae and Microbiota), 5. RCC (Roscoff Culture Collection) and 6. NA, Not available

### Tools

The database analysis portal provides access to the CKB BLAST tool, as well as tools for pattern and fuzzy searches, and restriction digestion.

The CKB BLAST tool can be used to compare nucleotide or protein sequences, to identify members of gene families, and to infer functional and evolutionary relationships between sequences.

**Fig 2:**
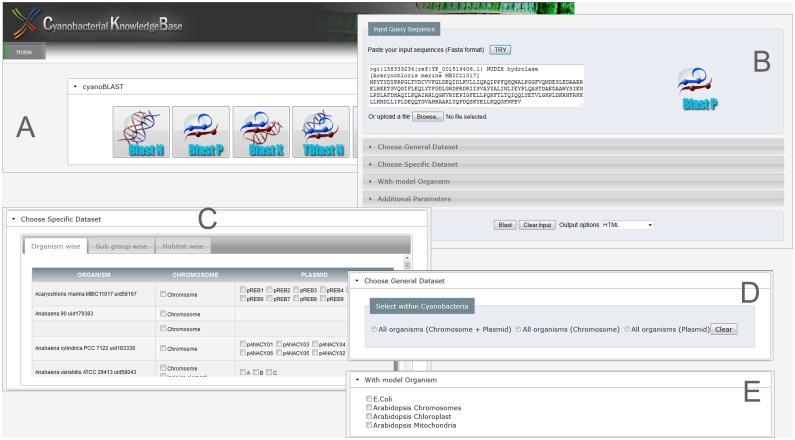
CKB BLAST tool. The BLAST analysis tool compares sequence within cyanobacterial species and with *E*. *coli* and *Arabidopsis*. It consists of A, B, C, D and E divisions where A: denotes different programs of BLAST tool, B: the sequence input page, C: option to select the individual or an order wise cyanobacterial dataset, D: choice to choose genomes of 74 cyanobacterial genomes fully or only chromosomes or only plasmids for analysis and E: option to choose *E*. *coli* and /or *Arabidopsis*.

Users are provided with several customized databases for similarity searches within the CKB BLAST analysis tool. This includes a database with information on all cyanobacterial chromosomes and plasmids. The users have the freedom to restrict their analysis to either chromosomes or plasmids. Furthermore, CKB provides databases that allow users to compare individual organisms, multiple organisms and orders also (Fig 2).

As cyanobacteria are prokaryotic photosynthetic organisms, a model prokaryotic genome (*E*. *coli*) and a photosynthetic eukaryotic genome (*Arabidopsis*) are included for advancing comparative analysis.

In addition, pattern and fuzzy search tools are available to help in identifying the patterns present in different cyanobacterial genomes. Furthermore, the restriction digestion tool helps to identify restriction sites within the sequences.

### Searching and browsing through the database

**Fig 3:**
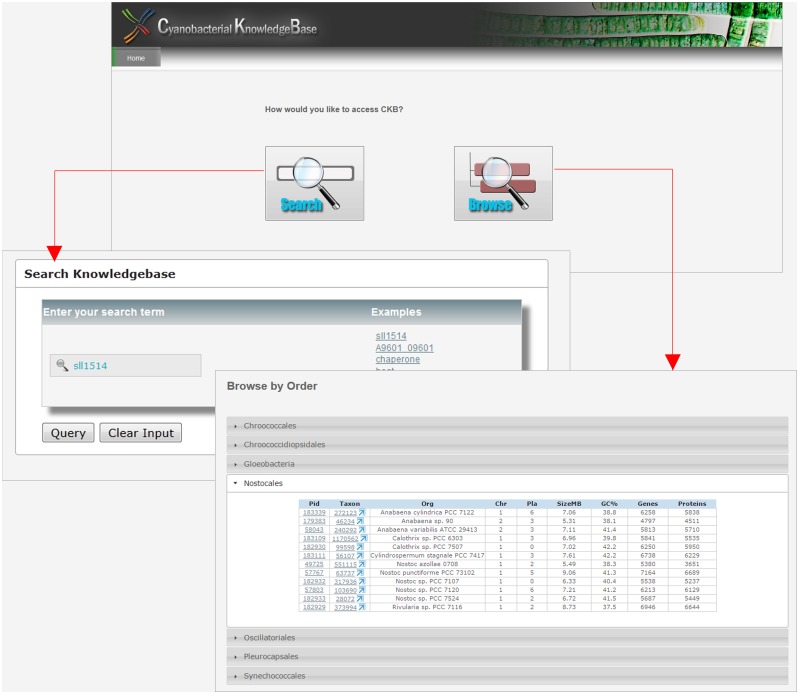
Search and browse tool. The CKB search tools allow to search by keywords or accession and browse tool, through which users can peruse to individual organism.

The Cyanobacterial KnowledgeBase consists of information related to 74 fully sequenced cyanobacterial species of seven orders, namely Chroococcales, Chroococcidiopsidales, Gloeobacteriales, Nostocales, Oscillatoriales, Pleurocapsales and Synechococcales. The browse option helps with orientation and navigation through the species under each order (Fig 3). The species report can be reached from the “Browse by Order” option, which provides brief information about the species, taxonomy, morphological features, genome status, and its genome details.

**Fig 4:**
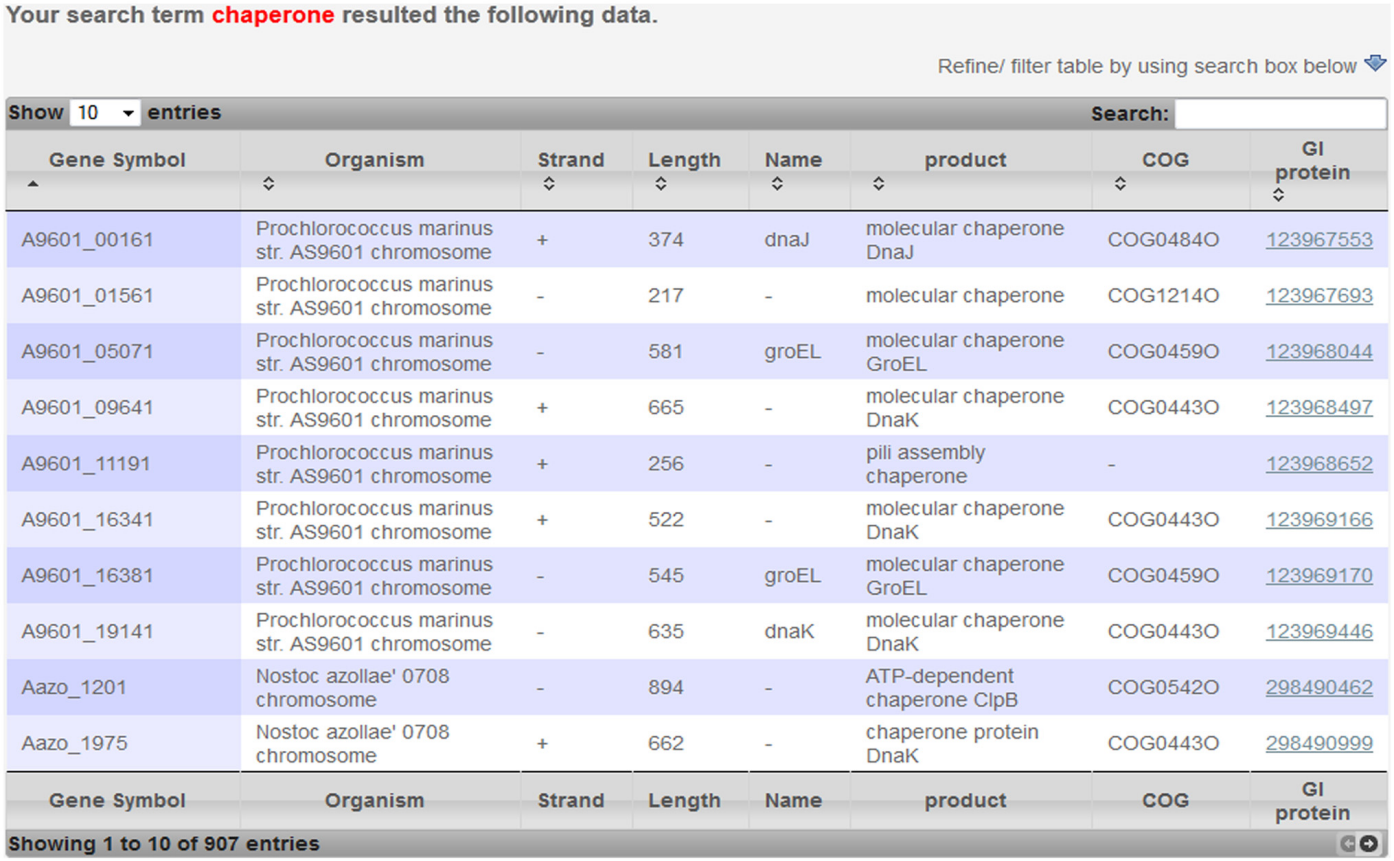
Search results. Results for keyword "Chaperone" showing 907 entries.

The search tool can also be used to retrieve information related to specific genes, functions, or keywords, etc. An example search result for a query keyword "Chaperone" returned 907 entries (Fig 4)

### Proteome profiling

The complete gene set of each genome can be accessed under the proteome profiling from the “Species reporter” tool. The table provides a complete gene list with PID gene name (locus_tag), synonym, product name, strand, start and end, length, COG (Clusters of Orthologous Groups) id and GI (Genbank) accession number. Furthermore, the search tool within the table provides an option to search and retrieve the results by specific keyword.

### Gene report

**Fig 5:**
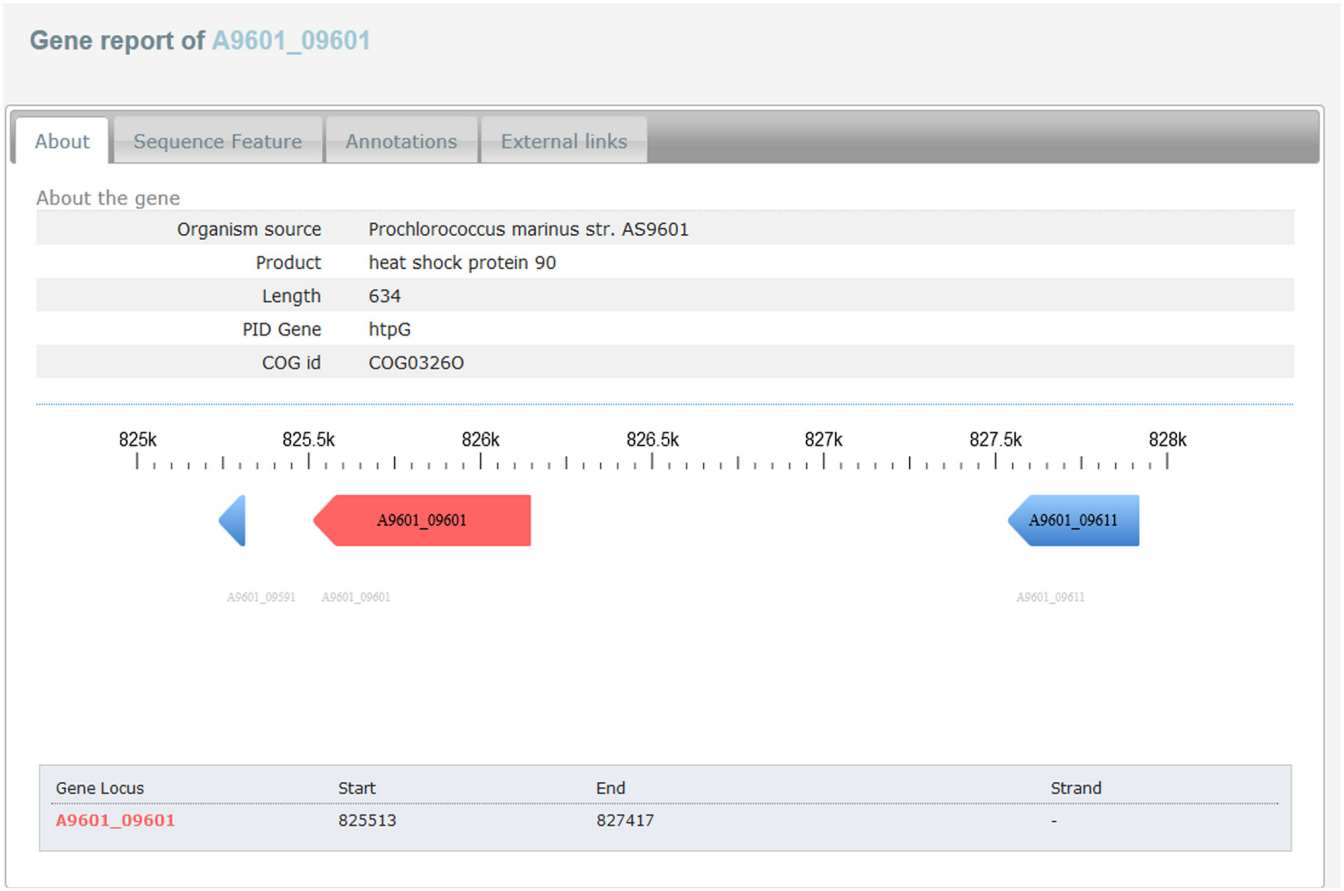
Gene report-About section. About section of the gene provides brief information about the gene of interest and genes located either side of the gene.

**Fig 6:**
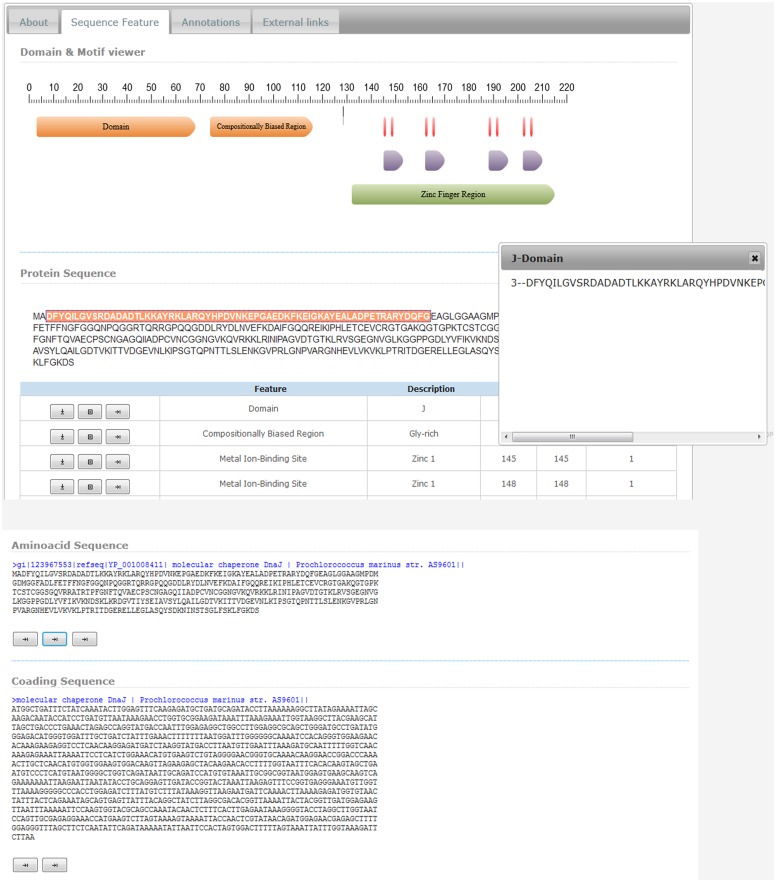
Sequence features. Graphical and tabular display of sequence features.

Information related to each gene is displayed under five sections. The ‘details section’ provides brief information related to the gene, and allows the user to navigate to the nearest genes present on either side of the gene of interest (Fig 5). The ‘sequence feature section’ provides domains, repeats, motif, and binding site information in both graphical and tabular form (Fig 6). The FASTA format of protein and nucleotide sequences are provided at the bottom section with direct links for BLAST analysis. The ‘annotation section’ displays the functions of the gene with gene ontology and UniProt keywords. The last two sections provide links to other external databases and list of homologous proteins respectively.

### Review of other related databases and web-resources

The rapidly increasing genomics and proteomics data due to advancements in high throughput data generation has created a need for enhanced data management to empower basic and applied research in cyanobacteria. Many web-based databases and community resources have been created specifically for cyanobacteria to facilitate systems biology analysis using these large data. Table 2 provides the list of databases summarized by Hernández-Prieto et al. [7] which has analytical tools along with the additional web resources and databases that are currently available.

**Table 2:** List of other databases or web-resources available as at May-2015.

Name	Database/Web resource	Details	Tools	Link to website
CyanoBase	Database	Database of genetic data of about 39 species	BLAST2, KazusaMart	http://genome.microbedb.jp/cyanobase/
CYORF	Database	Contains ORF list of about 33 species	BLAST, FASTA	http://cyano.genome.ad.jp/
CyanoBIKE	Database	A web-based programmable knowledge base for genomic, metabolic, and experimental data	In built and custom tools	http://biobike.csbc.vcu.edu/
cTFbase	Database	Database of transcription factors in the cyanobacterial genomes	BLAST, Multiple sequence alignment tool	http://bioinformatics.zj.cn/cTFbase/DatabaseLink.php
CyanoPhyChe	Database	Contains data related to physico-chemical properties, structure and biochemical pathway information of cyanobacterial proteins	NA	http://bif.uohyd.ac.in/cpc/
CyanoClust	Database	Database of homologous proteins in cyanobacteria and plastids	BLAST	http://cyanoclust.c.u-tokyo.ac.jp/
CyanoEXpress	Database	Used for examination and visualisation of gene expression changes for various experimental or genetic manipulations from numerous transcriptome studies	NA	http://cyanoexpress.sysbiolab.eu/
CyanoLyase	Database	A manually curated sequence and motif database of phycobilin lyases and related proteins	BLAST, Pattern matching tool (Protomatch)	http://cyanolyase.genouest.org/
Cyanorak	Database	Information system of clusters of orthologous sequences from marine picocyanobacteria	Blast	http://www.sb-roscoff.fr/cyanorak
SynechoNET	Database	Integrated protein-protein interaction database of *Synechocystis*	NA	http://bioportal.kobic.kr/SynechoNET/
ProPortal	Database	A resource for integrated systems biology of isolates of *Prochlorococcus*	NA	http://proportal.mit.edu/
CyanoNews	Web resource	Newsletter intended for cyanobacteriologists	NA	http://www.vcu.edu/cyanonews/
Cyanosite	Web resource	A general webserver for cyanobacterial research	NA	http://www-cyanosite.bio.purdue.edu/
CyanoData	Web resource	A database for methods for cyanobacterial bloom management	NA	http://www.cyanodata.net/faq.php
CyanoDB	Web resource	A taxonomic database of cyanobacterial genera.	NA	http://www.cyanodb.cz/

The most comprehensive and widely used web based database is CyanoBase [8], which contains currently sequenced and annotated genome sequences, along with gene annotations and information related to various mutations involved in 39 species of cyanobacteria. It also includes tools such as BLAST for genes and genome similarity searches and KazusaMart which can be used to convert identifiers from one format to different formats. CYORF is another community annotated database that provides the open reading frame (ORF) list for approximately 33 genomes along with data from KEGG and DBGET at the GenomeNet, Pfam and Prosite motifs, predicted localization sites and protein 3D structures and tools to search for similar sequences [9]. CyanoBIKE is an instance of BioBike which provides web-based programmable knowledge base for genomic, metabolic and experimental data specifically for cyanobacteria. It has the collection of different datasets along with built-in tools for analysis, which require some basic programming skills for its application [10].

Apart from the above three generalized cyanobacterial databases, there are a few more databases which are developed specifically for a particular species or a group of cyanobacteria, which includes Cyanorak [11], SynechoNET [12] and ProPortal [13]. These are dedicated resources with annotations for orthologous sequences of marine picocyanobacteria, protein-protein interaction data for *Synechocystis*, and information related *Prochlorococcus* isolates respectively.

Additionally, many specialized databases that are available focusing on specific protein class or property exclusively for cyanobacterial species. It includes cTFbase, a database containing transcription factors [14], CyanoPhyChe, which contains physico-chemical properties of cyanobacterial proteins [15], CyanoClust, which includes homolog groups in cyanobacteria and plastids produced by the program Gclust [16], CyanoEXpress, with curated genome-wide expression data [17] and CyanoLyase, a database of phycobilin lyase sequences, motifs and functions [18].

Along with these online databases, CyanoNews [19], Cyanosite [20], CyanoData [21] and CyanoDB [22] are the major web resources that provide the basic information about cyanobacteria, current happenings in cyanobacterial research, the methods used in cyanobacteriology, bibliography archive, research groups involved in cyanobacterial research, etc. that are extensively referred by cyanobacteriologists.

CKB, the present available database has incorporated all 74 currently fully sequenced genomes of cyanobacteria, including customized tools for inclusive analysis of these genomes. The tool also helps in interpreting newly sequenced genomes by comparing them with the previously annotated cyanobacterial and/or other model organism genomes. The flexibility of defining datasets by either organism or order, or as whole genome or plasmids, helps the user to segregate their search and its results according to their specific needs. An additional significant characteristic is the inclusion of the model prokaryotic genome (*E*. *coli*) and presence of a photosynthetic eukaryotic genome (*Arabidopsis*), which further assists in comparative sequence analysis thereby making CKB a unique and beneficial resource for cyanobacterial genome analysis.

### Future Prospects

It is planned to improve and update the content of the database of CKB in the following aspects. First, gene information will be enriched by adding experimentally proven results related to biological functions, expression, and protein-protein interactions by manually curating the data from peer reviewed literature. In addition, we intend to include or develop further analysis tools to support the analysis of cyanobacterial genomes. The necessary efforts will also be made to ensure the database as user-friendly and efficient as possible, using the reflection and feedback from users of the first version of CKB to guide our efforts.

## Conclusions

Here we present CKB as a knowledge database for the cyanobacteriologists. CKB provides access to information related to fully sequenced genomes and can be utilized for analysis and retrieving information. The CKB database website is freely accessible as a web application at: http://nfmc.res.in/ckb.

## Materials and Methods

### Data Collection and Organization

The complete genomes of 74 cyanobacteria were downloaded from the NCBI ftp site and their accession numbers are listed in S1 File [23]. Sequence features, annotations, and external links were downloaded from the UniProt database in xml format for each gene [24]. All the downloaded data from NCBI and UniProt databases were converted into csv format and uploaded into a SQL database. The full schema of the database is included as the S1 Fig.

### Web Interface and Application

CKB is built on a 64 bit CentOS (version 5) server running WAMPSERVER (V2.2d), which integrates the Apache HTTP Server (V2.2.21) with PHP (V5.3.10) and the MySQL Server (V5.5.20). Complete data related to the sequence and annotations are stored in a MySQL database. The database is designed using PHP, with jQuery JavaScript Library (V1.10), and Cascading Style Sheets (CSS) for the web interface. In addition, a simple gene browser in HTML5 is incorporated into the gene report page, which is provided by Chase Miller [25]. The BLAST 2.2.29+ tool is downloaded from NCBI ftp and pattern and fuzzy search tool and the restriction digestion tools are downloaded from Sequence Manipulation Suite [26–27]. The web server and all information parts of the database are hosted at NFMC portal www.nfmc.res.in.

## Supporting Information

S1 Fig Database schema. (TIF)

S1 File List of RefSeq accession numbers. (DOCX)
