# Supplementary figures and images for: Cyanobacterial KnowledgeBase (CKB), a Compendium of Cyanobacterial Genomes and Proteomes
Source: PLoS One. 2015 Aug 25;10(8):e0136262. doi: 10.1371/journal.pone.0136262 (PMC4549288; doi:10.1371/journal.pone.0136262)

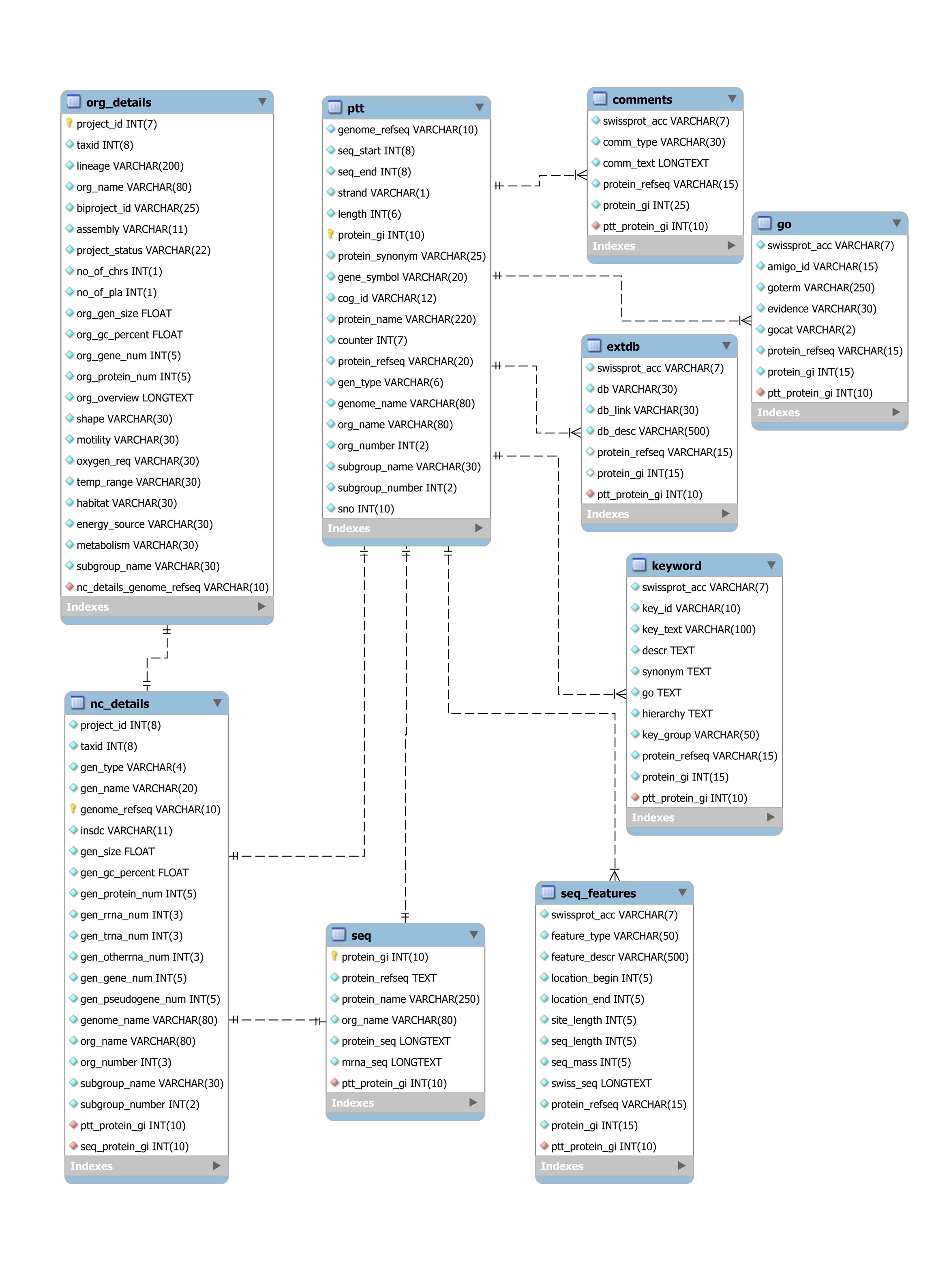

Supplement: S1 Fig — (TIF) [file pone.0136262.s001.tif]
