# Supplementary material for: Cyanobacterial KnowledgeBase (CKB), a Compendium of Cyanobacterial Genomes and Proteomes
Source: PLoS One. 2015 Aug 25;10(8):e0136262. doi: 10.1371/journal.pone.0136262 (PMC4549288; doi:10.1371/journal.pone.0136262)
Supplement: S1 File — (DOCX) [file pone.0136262.s002.docx]

Accession Numbers

NC_009925, NC_009926, NC_009927, NC_009928, NC_009929, NC_009930, NC_009931, NC_009932, NC_009933, NC_009934, NC_019771, NC_019772, NC_019773, NC_019774, NC_019775, NC_020157, NC_020056, NC_019427, NC_019439, NC_019429, NC_019428, NC_019440, NC_007413, NC_014000, NC_007411, NC_007410, NC_007412, NC_016640, NC_019751, NC_019752, NC_019727, NC_019728, NC_019682, NC_019697, NC_019698, NC_020053, NC_019695, NC_019696, NC_019699, NC_019753, NC_019756, NC_019737, NC_019734, NC_019754, NC_019755, NC_019735, NC_019736, NC_019733, NC_019776, NC_019777, NC_019778, NC_019675, NC_010546, NC_010547, NC_010539, NC_010541, NC_010542, NC_010543, NC_011729, NC_011738, NC_011737, NC_011730, NC_011732, NC_011733, NC_011734, NC_011884, NC_011880, NC_011885, NC_011882, NC_014501, NC_014503, NC_014504, NC_014533, NC_014535, NC_014534, NC_014502, NC_011726, NC_011721, NC_011723, NC_011727, NC_013161, NC_013160, NC_013163, NC_013167, NC_013168, NC_019757, NC_019758, NC_019744, NC_020050, NC_019780, NC_019703, NC_022600, NC_005125, NC_019745, NC_019746, NC_019747, NC_019759, NC_020051, NC_019779, NC_019683, NC_019738, NC_019762, NC_019739, NC_019740, NC_019741, NC_019760, NC_019742, NC_019761, NC_019743, NC_010296, NC_014248, NC_014249, NC_014250, NC_010628, NC_010631, NC_010632, NC_010630, NC_010633, NC_010629, NC_019676, NC_003272, NC_003276, NC_003240, NC_003273, NC_003270, NC_003267, NC_003241, NC_019684, NC_019685, NC_019677, NC_019693, NC_019700, NC_019694, NC_019729, NC_019764, NC_019732, NC_019731, NC_019763, NC_019730, NC_019689, NC_008816, NC_009976, NC_009840, NC_009091, NC_008820, NC_007577, NC_005071, NC_008817, NC_008819, NC_007335, NC_005042, NC_005072, NC_019701, NC_019690, NC_019678, NC_019679, NC_019686, NC_019748, NC_019749, NC_019750, NC_019765, NC_019766, NC_020052, NC_006576, NC_007604, NC_007595, NC_008319, NC_007516, NC_007513, NC_007776, NC_007775, NC_019680, NC_019681, NC_010475, NC_010476, NC_010477, NC_010478, NC_010479, NC_010480, NC_010474, NC_019702, NC_019691, NC_019692, NC_009482, NC_009481, NC_005070, NC_017277, NC_000911, NC_005230, NC_005231, NC_005229, NC_005232, NC_020286, NC_020290, NC_020296, NC_020298, NC_020287, NC_020288, NC_020289, NC_020297, NC_017038, NC_017052, NC_017039, NC_004113, NC_023033, NC_008312
